# Using Organoids to Tap Mammary Gland Diversity for Novel Insight

**DOI:** 10.1007/s10911-024-09559-z

**Published:** 2024-03-28

**Authors:** Gat Rauner

**Affiliations:** Department of Developmental, Molecular & Chemical Biology, Tufts University School of Medicine, Boston, MA 02111 USA

**Keywords:** Organoid Technology, Lactation, Animal Models, Ex-Vivo, Evolution of Development

## Abstract

This article offers a comprehensive perspective on the transformative role of organoid technology on mammary gland biology research across a diverse array of mammalian species.

The mammary gland's unique development and regenerative capabilities render this organ an ideal model for studying developmental evolution, stem cell behavior, and regenerative processes. The discussion extends to the use of cross-species mammary organoids to address key biological inquiries in evolution, tissue regeneration, cancer research, and lactation, highlighting the limitations of traditional mouse models and the benefits of incorporating a more diverse range of animal models.

Advances in organoid biology have been critical in overcoming ethical and practical constraints of in-vivo studies, especially in human research. The generation of human and mouse mammary organoids that faithfully recapitulate in-vivo tissues marks a significant stride in this field. Parallel capabilities are now emerging for other mammals, as well.

Utilizing mammary organoids from various species has the potential to make invaluable contributions to our understanding of mammary gland biology, with implications for regenerative medicine, cancer research, and lactation studies, thereby contributing to advancements in human health, agriculture, and nutrition science.

## Introduction

**Fig. 1:**
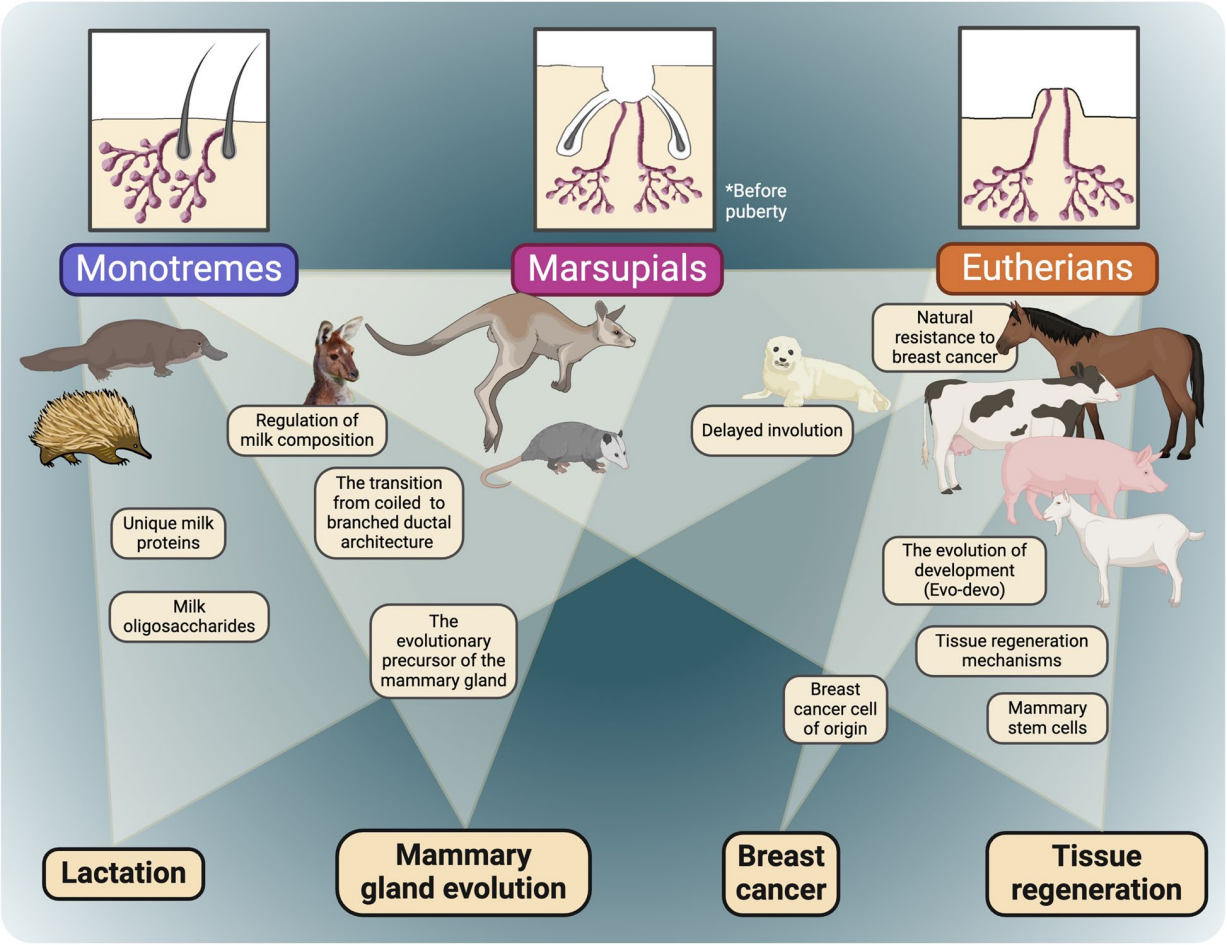
Schematic outline of the different research questions that using cross-species mammary gland organoids could facilitate

This perspective article aims to discuss the value of studying the biology of mammary glands across a variety of mammalian species, to delineate the potential benefits of this research direction, and to suggest that advances in organoid biology can remove past barriers and increase access to diverse animal models. The first section will discuss the unique potential of the mammary gland as a tissue and organ to explore the evolution of development. The following sections will delve into the specific biological questions that can be answered by using cross-species mammary organoids in evolution, as well as in the fields of stem cells and regeneration, breast cancer, and lactation (Fig. 1).

### The Unique Case of the Mammary Gland

The mammary gland is unique in its developmental timeline and regenerative capacity. During embryonic development, a rudimentary gland is established and remains quiescent until puberty. Upon puberty, the gland expands and matures to its adult form. The adult mammary gland responds to hormonal stimuli with each menstrual or estrus cycle and is capable of further maturation and milk production during reproductive cycles. Therefore, the mature mammary gland exists in a state of readiness, prepared to respond to the physiological stimulus of reproduction, with robust expansion and differentiation, culminating in the emergence of an organ that is essential for the survival of the species, the lactating gland. All stages of mammary gland development, maturation, and regeneration are thus under strong evolutionary selection, manifested by many conserved characteristics across mammals, as well as many unique adaptations that evolved in response to specific pressures. All this makes the mammary gland a paradigmatic model organ to explore the mammalian evolution of tissue development, regeneration, and adult stem cells.

The state of readiness that the mammary gland exists in for extended periods during an organism’s life requires the maintenance of a pool of regenerative cells to facilitate the vast expansion that occurs upon pregnancy and lactation. However, as many of the readers will know, despite decades of studies aimed at elucidating the identity of mammary stem cells, they have yet to be conclusively identified [1]. This contrasts with several other epithelial tissues like the skin and intestinal epithelium, where stem cells constitutively regenerate the epithelium and have long been identified, located, and characterized [2, 3]. A possible reason for the elusiveness of mammary stem cells may be the gland’s “state of readiness”. Indeed, we do not know whether a set of designated cells exists quiescently in the tissue and is activated upon hormonal stimulation, or whether they are designated as stem cells only upon receipt of the stimulus and/or tissue changes that follow. There is a lot of evidence suggesting that mammary epithelial cells possess a large degree of plasticity, and that context, in the form of the extracellular matrix, neighboring cells, hormonal, and other stimuli determines their cell fate decisions [4–8]. This plasticity may be the reason that the regeneration of this tissue is still not entirely understood, and it may hold promise for regenerative medicine if the controls of cell fate are deciphered in this unique tissue, in addition to its obvious contribution to a better understanding of developmental pathologies of the breast. Understanding the mechanisms of mammary gland development and regeneration may also result in a better understanding of breast tumorigenesis, particularly the early stage of normal cells turning malignant, which is also not entirely understood [9].

In addition to mammary tissue regeneration and tumorigenesis, other aspects that are not yet understood concern the regulation of lactation, and particularly milk composition. We know that milk composition changes with offspring age, and varies between individuals and between species, but we do not have a clear understanding of how this process is regulated at the cell and tissue level.

Most of the studies into these aspects of mammary gland physiology have been done in human tissues, cells, and cell lines, or rodent models – particularly mice. Mouse models offer the possibility to conduct experiments in-vivo and modify genes. However, the mouse mammary gland does not recapitulate many aspects of the human breast, important among which are the ductal-lobular architecture and the stromal composition [10]. While mouse models continuously evolve with emerging genetic and imaging tools, they cannot overcome this inherent limitation.

Expanding the availability of animal models could tap knowledge and understanding that have been out of reach, by enabling the exploration of phenotypes that may not exist in rodent models and indeed even in humans. This perspective will provide several examples that have already been recognized and are studied by a small number of groups around the world. These examples are likely just the tip of the iceberg, as mammals are a very large and diverse group of species, with varying reproduction strategies, that have evolved to populate nearly every corner of the earth.

### The Potential of Organoid Technology

Organoids, miniature versions of tissues and organs that grow in 3D culture, serve as an in-vitro alternative where in-vivo experimentation is unavailable, such as in humans. Recent advances in organoid technology resulted in the generation of human and mouse mammary organoids that not only recapitulate key aspects of the in-vivo tissue but also, importantly, the process of its development and maturation [11–18]. The status of mammary organoid technology has been reviewed elsewhere (see [15, 19–22]) and is progressing rapidly.

The next-generation version of human breast organoids grows in a biomimetic extracellular matrix (ECM) that incorporates, in addition to type I collagen, key components of human breast ECM: hyaluronic acid, fibronectin, and laminin [23]. This ECM enables the generation of human breast organoids that effectively recapitulate their in-vivo counterparts in multiple aspects: cell population heterogeneity, morphology and anatomy, and response to hormones [11, 23]. Importantly, they also include an inductive mesenchyme, an often-neglected component in 3D organoid models, but one that is essential to its recapitulation of normal physiology in the mammary gland and other tissues [24, 25]. Organoids can be monitored in real-time using long-term confocal live imaging, and their development from a single cell can be recorded and analyzed. This technology enables high throughput analysis (96-well) and is amenable to genetic modifications using lentiviral transduction. Organoids from single cells have the potential to capture detailed organogenesis dynamics and cellular diversity over time. Recently, mouse mammary organoids were also taken to the next level, showcasing additional developmental phases, including involution and post-involution regeneration [12].

Mammary organoids can be generated from cryopreserved primary cells, and do not require consistent donor availability. Therefore, similar generation of organoids from additional species opens the possibility of studying mammary gland physiology in species that were previously unavailable for experimental studies, such as large, rare, and wild species, and those that are unsuitable as lab animals for a variety of reasons. Interestingly, some studies report the generation of mammary gland organoids from induced pluripotent stem cells (iPSCs) [26, 27], introducing the possibility of generating mammary gland organoids from non-mammary tissues.

Recently, using the biomimetic ECM system, mammary gland organoids were generated from 9 mammalian species, including 8 eutherian mammals (cat, cow, ferret, goat, hamster, pig, rabbit, and rat) and one marsupial (opossum) [28]. These organoids have a complex branched morphology, rather than rudimentary spheroid or budding spheroids that developed within other matrices [29–35]. Further studies will determine to which capacity they recapitulate the cell types in the original tissue, and whether adjusting the ECM can optimize the organoids in a species-specific manner.

Notably, organoids have limitations. They cannot replace in-vivo tissue in every aspect, and the currently existing models are far from a perfect mimic of tissue complexity. As such, results should always be interpreted with caution and aspire for in-vivo validation where possible. That said, organoid technology is evolving and continuing to incorporate more bio-mimetic features such as vasculature, combinations of complementary organ systems and engineered extracellular matrices. 

The next sections will discuss the potential of mammary organoids to facilitate research related to mammary gland evolution, breast cancer, stem cells and tissue regeneration, and lactation biology.

### Mammary Gland Evolution

Mammals can be classified into three primary groups: monotremes, marsupials, and eutherians. Monotremes are the most ancient among extant mammals: they diverged from other mammalian groups approximately 190 million years ago [36]. Monotremes lay eggs and lactate the altricial hatched puggles during the vulnerable initial stages of their life. Their mammary glands consist of a patch of specialized hairs, and each hair is associated with an individual gland [37]. Milk is secreted to the skin surface; there is no nipple. This structure resembles the hair-associated apocrine gland, which is thought to be the evolutionary precursor to the mammary gland [38]. It is therefore obvious that monotremes are key species in research that aims to explore the evolution of the mammary gland, and indeed of mammalian evolution and the emergence of mammals. However, access to live monotremes is challenging: the monotreme species that exist today include only the platypus and four species of echidna. The platypus (Ornithorhynchus anatinus) is geographically restricted to eastern Australia and is listed as “Near Threatened” in the International Union for Conservation of Nature (IUCN) Red List of Threatened Species [39]. Of the four echidna species, only the short-beaked echidna (Tachyglossus aculeatus) has a wide distribution across the Australian continent, while the others are restricted to Indonesia and/or Papua New Guinea and are listed as “critically endangered” or “vulnerable” in the IUCN Red List [40]. Monotremes do not breed easily in captivity, and they are all, including the more widespread echidnas, considered protected species in Australia [41]. Monotreme mammary organoids have the potential to overcome the limitations of access to monotreme tissue and can be an important resource in addition to existing resources of non-living samples and other data, such as the Monotreme Resource Center at the University of Adelaide [42]. Among the findings resulting from the exploration of the monotreme mammary gland is the discovery of monotreme-specific milk proteins with antibacterial activity: MLP (monotreme lactation protein) and EchAMP (echidna antimicrobial protein), which authors suggest could be used as a potential prophylactic against mastitis in dairy animals [43–47].

Marsupials, which diverged from Eutherians approximately 160 million years ago, present an intermediate form of a mammary gland between the primitive monotreme form and the Eutherian ones. Like monotremes, marsupials often feature in evolutionary research, including research into the adaptation of genes and pathways throughout evolution [48–50]. The marsupial mammary gland develops in association with hair, but the hair disappears in the adult gland [51, 52]. Marsupials produce specialized milk that compensates for their short gestation periods and lack of substantial placentation. An intriguing aspect of marsupial lactation found in macropods such as kangaroos and wallabies, is known as “asynchronous concurrent lactation” (ACL) [53–55]. ACL allows a mother to produce two types of milk at the same time, each with a composition suited to the nutritional needs of her offspring at different developmental stages. Species with ACL provide a unique opportunity to study the local regulation of milk composition, isolated from systemic effects [56–58].

Lastly, eutherians represent the largest and most diverse group, with wide physiological variations and habitats across ecosystems globally. Eutherian mammary glands have evolved to cater to the specific needs of their species – from the number of teats, the shape and size of the gland, to distinct lactation modes that range from continuous to intermittent, short to long, and milk composition variations between species, and throughout the lactation period.

As mentioned, branched organoids from 8 eutherian mammals and one marsupial have been generated recently [28], using a collagen-based matrix that had been originally developed for culturing human breast organoids [23]. Organoids from different species manifest varied architectures under these conditions and require varied media supplements to grow and branch. Optimizing organoid growth for each species is an important goal for improving the technology and the model, but it can also have additional value if it reveals an underlying molecular or cellular mechanism that is key for certain aspects of development. For example, branching of mammary organoids of some species (cow, goat, rabbit, and opossum) requires the addition of ROCK inhibitors to the culture media. This finding highlights a previously observed role of ROCK proteins in the process of branching, which is understudied, and its mechanism unresolved. It also presents a comparative animal model in which this role can now be studied mechanistically. Furthermore, using organoids from evolutionarily distant branches of mammals, such as marsupials and monotremes can reveal how these developmental mechanisms evolved. Finally, organoids can be used to study the functional consequences of specific genetic changes or adaptations within a species.

The next sections will discuss a partial list of specific cases where mammalian variability is an opportunity for comparative research and has the potential to yield impactful findings and expand knowledge across several fields of study.

### Breast Cancer Research

Despite years of research, some fundamental questions about breast cancer remain unanswered. In particular, we still don’t know what the breast cancer cells of origin are in humans, and what causes those cells to undergo malignant transformation and form a cancerous tumor [59, 60]. The early events of breast cancer remain elusive despite many models of breast cancer in mice, where its cells of origin have been investigated [61–65]. Since cancer of the mammary gland does not develop spontaneously in non-transgenic mice in the same manner it does in humans, but rather results from a retroviral infection which is also transmitted in the milk [66–69], mouse models of breast cancer rely on genetic modifications and mutations to cause mammary tumors in-vivo or PDX (patient-derived xenograft) models where human tumor cells are transplanted in mice. Both approaches are inherently limited in their ability to address the natural occurrence of breast cancer and tumors of the mammary gland in species, including humans, where mammary tumorigenesis is not necessarily virally induced.

Incredibly, it is still not common knowledge that some mammals rarely, if ever, develop cancer of the mammary gland [70–72]. These mammary cancer-resistant mammals include pigs, cows, sheep, goats, and horses, and there may be additional ones for which we do not have reliable data regarding mammary cancer occurrence. This phenomenon received some attention years ago but later faded from research focus and it still remains unknown what makes some species resistant to this type of cancer, which is so prevalent and fatal in humans [73]. While billions of dollars are rightfully dedicated to research aimed at breast cancer prevention, diagnosis, and treatment, essentially none of these fund are invested in taking a deep look at a phenomenon that has the potential to answer long-standing, core questions about breast cancer. But funding is not the only obstacle: the above list of species is not easily accessible to most breast cancer labs, which are mostly located in hospitals, medical schools, and university departments of life sciences, not in veterinary schools, agricultural institutions, or farms. Organoids have the potential to change the accessibility of these species to breast cancer research labs.

Several hypotheses can explain the rarity of mammary tumors in some species. It has been hypothesized that differences in DNA damage response between mammary cancer-resistant and susceptible species, and particularly in the threshold of DNA damage that leads to apoptosis, could explain the survival vs. elimination of damaged cells in susceptible vs. resistance species, respectively [74–76]. Limited data supporting this hypothesis was collected in 2D models of mammary epithelial cells derived from relevant species but was not verified in 3D or in-vivo. Alternatively, it can be hypothesized that differences in estrogen exposure during the estrus cycle might explain the difference between species. Parity and lactation have been suggested (but not confirmed) to confer the resistance of dairy animals, but this explanation may not apply to non-dairy animals such as pigs and horses [73].

While organoids cannot replace in-vivo experiments and validation, they do offer more than an in-vitro organ to experiment on: the ability to track the development of organoids from single cells to a mature tissue offers a window to developmental processes that may be at the basis of cancer susceptibility. Cancer and development are tightly linked, and organoids offer the potential to explore both in species that may offer novel insight into fields that could benefit human health, particularly for answering questions that the existing methods and models have not been able to answer.

### Stem Cells and Tissue Regeneration

As mentioned above, following embryonic and pubertal development, the mammary gland regenerates with every cycle of reproduction and lactation. However, the cells that produce the regenerated tissue, the adult stem cells of the mammary gland, have not been clearly identified – not in mice, nor in any other species, including humans [1]. A fundamental leap in our understanding of mammary stem cells and their capacity to contribute to the regeneration of this tissue came over a decade ago: lineage tracing experiments in mice showed that while removing cells from their native environment can render them able to regenerate all the lineages of the gland (as is the case with cleared fat-pad transplantations), this is likely not how the gland regenerates in-vivo [77]. Instead, the post-natal gland is regenerated by lineage-restricted progenitors, and not by bi-potent stem cells, as was previously thought. While these lineage-tracing experiments had limitations due to the genetic models they use, and different models and analysis methods yielded complex and conflicting results [78–83], they remain the most direct way to observe stem cell fate in situ. These pivotal findings in mice were not confirmed in humans or any other mammals, due to the inaccessibility of lineage-tracing experiments in these species. Interesting clonal tracing in the human breast tissue, based on somatic mutations in mitochondrial enzyme cytochromecoxidase (CCO), provide indications that a bi-potent stem cell may exist in the adult breast, and that it may reside in the luminal compartment [84]. Given the evidence of extensive plasticity of mammary epithelial cells, and the strong pressures to always maintain its regenerative capacity in a state of readiness, these questions are key to understanding the regeneration strategy of this tissue.

Here, too, organoids can be an experimental model to answer these questions. First, we can design genetic tools that will allow lineage tracing in organoids. This will allow us to conduct lineage tracing on human breast tissue. Important in this context is the fact that the next-generation breast organoids contain a stroma, which may play a key role as part of the microenvironment that controls stem cell fate. Another key feature relevant is the recapitulation of multiple cell types within the organoid, similar to the human breast. Second, we can compare the regeneration capacity of organoids from different species. One of the relevant features of organoids for this purpose is that they maintain a relatively constant pool of regenerative cells within the organoid [11]. This was observed when breast tissue organoids were formed from primary cells derived from 12 donors. In the first generation of organoids, there was significant variability between patient samples in the number of organoid-forming cells, but when a second generation of organoids was formed by cells dissociated from organoids, the variability was much lower between patient samples and averaged 1 organoid per 100 organoid-derived cells. This implies that the pool of cells with the potential to regenerate the tissue is controlled within the organoid. Organoid technology can lead to the identification of those cells within the organoid and a better understanding of how their plasticity and fate are controlled.

### Lactation Biology

The study of lactation biology encompasses the initiation, duration, and cessation of milk production, as well as the composition of milk, including milk bio-actives like oligosaccharides. Mammary organoids offer the possibility to explore these aspects across mammals.

The initiation of lactation, a complex process influenced by hormonal changes, can be closely examined and monitored in organoids. This can be achieved by controlling conditions such as hormone levels, the composition and mechanical properties of the extracellular matrix, and importantly by the ability to genetically modify the organoids. These capabilities are not readily available even where access to in-vivo experimentation is easy, and they are certainly not possible in species where this access is restricted, including in humans. Such studies can have implications for understanding and treating lactation problems in humans and livestock.

The cessation of lactation and the process of involution where the mammary gland returns to its pre-lactation state are equally vital aspects, that, as was recently shown, can also be studied in mammary organoids [12]. The mechanisms behind the induction of involution are not entirely clear [85]. The first, reversible phase of involution, which lasts for 48 hours in mice, is STAT3 dependent [86]. Evidence from mouse experiments shows that milk stasis induces cell death through the uptake of milk fat globules by mammary epithelial cells. When delivered to the lysosomes, the free fatty acids make the lysosomal membrane permeable resulting in the release of cathepsins to the cytosol, followed by cell death [87, 88]. The next, irreversible phase of involution involves systemic hormonal signals [89]. However, there are species, such as the fur seal, where involution does not occur through weeks-long periods during which the mother is away foraging at sea and the pups are not lactating. Studying this phenomenon has led to important discoveries implicating the milk protein alpha-lactalbumin, which is missing in fur seals [90, 91]. Lack of or mutated alpha-lactalbumin leads to lactose-free milk and can result in delayed involution despite milk stasis. It was subsequently shown that alpha-lactalbumin is an apoptotic factor in mammary epithelial cells, suggesting that it plays a role in the natural process of mammary gland involution [92]. Access to in-vivo experiments that would follow up on these findings is understandably limited and is another example where mammary organoids can bridge the gap and allow further exploration of this phenomenon to gain a better understanding of the process of mammary gland involution, and the role of milk components in this process.

The composition of milk, including the presence of bioactive compounds like oligosaccharides, is a current area of study [93, 94]. Mammary tissue organoids can allow for the analysis of milk secretion and its compositional changes under various conditions. This is particularly important for understanding the nutritional and immunological aspects of milk and can lead to the development of improved infant formulas and dairy products. For example, marsupials have been the focus of studies related to milk composition because they secrete rich milk during early lactation, supporting the early development of offspring that are born underdeveloped following short pregnancies that lack significant placentation [95–98]. As mentioned above, marsupials are also capable of asynchronous concurrent lactation and are therefore a model to study tissue-local controls over milk composition.

Milk oligosaccharides are the third most abundant solid component of human milk [99] and are an important contributor to infant development and particularly neurodevelopment [94]. The composition and types of oligosaccharides differ greatly between species [93, 100–105], and it is hypothesized that milk oligosaccharide abundance and variety are important evolutionary contributors to the development of complex mammalian systems, with an emphasis on neural systems [106, 107]. The variability of milk oligosaccharides is not directly encoded in the genome but is generated in situ and impacted by the cellular context and factors such as hormones and enzymes involved in glycosylation pathways. Evidence is accumulating that mammary gland organoids are capable of secreting milk components, including milk proteins and lipids [15, 23, 26, 32, 108]. An organoid model that induces secretion of oligosaccharides can facilitate experimental research that would shed light on mechanisms that regulate the variety and abundance of milk oligosaccharides across mammals, with relevance to the evolution of mammals and lactation, and also to the mammary gland microbiome [109, 110].

In conclusion, mammary tissue organoids from various mammalian species are invaluable in advancing our understanding of lactation biology. They provide a versatile and controlled environment for exploring the complex biological processes involved in milk production, offering potential benefits in human health, agriculture, and nutritional sciences.

## Concluding Remarks

This article underscored the value of expanding research on mammary gland biology across diverse mammalian species using organoids. The mammary gland's unique developmental and regenerative characteristics position it as a good model for investigating fundamental biological questions in evolution, cancer, stem cell research, and lactation. Expanding the mammalian diversity available for research of mammary gland biology enriches the knowledge resources and will deepen our understanding of mammary gland evolution and function, stem cell dynamics, and tissue regeneration, with real potential for breakthroughs in breast cancer research, regenerative biology, and nutritional sciences.

## Data Availability

No datasets were generated or analysed during the current study.

## References

[1] Watson CJ (2021). How Should We Define Mammary Stem Cells?. Trends Cell Biol.

[2] Morris RJ (2004). Capturing and Profiling Adult Hair Follicle Stem Cells. Nat Biotechnol.

[3] Barker N (2007). Identification of Stem Cells in Small Intestine and Colon by Marker Gene Lgr5. Nature.

[4] Rauner G (2021). Breast Tissue Regeneration is Driven by Cell-Matrix Interactions Coordinating Multi-Lineage Stem Cell Differentiation Through DDR1. Nat Commun.

[5] Englund JI (2022). Laminin Matrix Adhesion Regulates Basal Mammary Epithelial Cell Identity. J Cell Sci.

[6] Kass L (2007). Mammary Epithelial Cell: Influence Of Extracellular Matrix Composition and Organization During Development and Tumorigenesis. Int J Biochem Cell Biol.

[7] Wicker MN, Wagner K-U (2023). Cellular Plasticity in Mammary Gland Development and Breast Cancer. Cancers.

[8] Naylor MJ (2005). Ablation of Beta1 Integrin in Mammary Epithelium Reveals a Key Role for Integrin in Glandular Morphogenesis and Differentiation. J Cell Biol.

[9] Amend SR (2018). Ten Unanswered Questions in Cancer: "If this is true, what does it imply"?. Am J Clin Exp Urol.

[10] Cardiff RD, Wellings SR (1999). The Comparative Pathology of Human and Mouse Mammary Glands. J Mammary Gland Biol Neoplasia.

[11] Rauner, G., et al., Advancements in Human Breast Organoid Culture: Modeling Complex Tissue Structures and Developmental Insights. bioRxiv, 2023: p. 2023.10.02.560364.

[12] Yuan L (2023). Reconstruction of Dynamic Mammary Mini Gland in Vitro for Normal Physiology and Oncogenesis. Nat Methods.

[13] Ganz HM (2021). Generation of Ductal Organoids from Normal Mammary Luminal Cells Reveals Invasive Potential. J Pathol.

[14] Linnemann JR (2015). Quantification of Regenerative Potential in Primary Human Mammary Epithelial Cells. Development.

[15] Sumbal J (2020). Primary Mammary Organoid Model of Lactation and Involution. Front Cell Dev Biol.

[16] Caruso M (2022). A Mammary Organoid Model to Study Branching Morphogenesis. Front Physiol.

[17] Charifou E (2021). A Robust Mammary Organoid System to Model Lactation and Involution-Like Processes. Bio-Protoc.

[18] Rosenbluth JM (2020). Organoid Cultures from Normal and Cancer-Prone Human Breast Tissues Preserve Complex Epithelial Lineages. Nat Commun.

[19] Mohan SC (2021). Current Status of Breast Organoid Models. Front Bioeng Biotechnol..

[20] Lee J (2023). Human Breast Organoid Models for Lactation Research. Reproduction Breed.

[21] Srivastava V (2020). Organoid Models for Mammary Gland Dynamics and Breast Cancer. Curr Opin Cell Biol.

[22] Lewis SM, Callaway MK, dos Santos CO (2022). Clinical Applications of 3D Normal and Breast Cancer Organoids: a Review of Concepts and Methods. Exp Biol Med.

[23] Sokol ES (2016). Growth of Human Breast Tissues from Patient Cells in 3D Hydrogel Scaffolds. Breast Cancer Res.

[24] Richards Z (2019). Prostate Stroma Increases the Viability and Maintains the Branching Phenotype of Human Prostate Organoids. IScience.

[25] Hofer M, Lutolf MP (2021). Engineering Organoids. Nat Rev Mater.

[26] Qu Y (2017). Differentiation of Human Induced Pluripotent Stem Cells to Mammary-Like Organoids. Stem cell Rep.

[27] Dai X (2022). Human Fibroblasts Facilitate the Generation of iPSCs-Derived Mammary-Like Organoids. Stem Cell Res Ther.

[28] Kim HY (2024). Expanding the Evo-Devo Toolkit: Generation of 3D mammary Tissue from Diverse Mammals. Development.

[29] Bartlett AP (2022). Establishment and Characterization of Equine Mammary Organoids using a Method Translatable to Other Non-Traditional Model Species. Development.

[30] Ellis S (2000). Growth and Morphogenesis of Epithelial cell Organoids from Peripheral and Medial Mammary Parenchyma of Prepubertal Heifers. J Dairy Sci.

[31] Arevalo Turrubiarte M (2016). Phenotypic and Functional Characterization of Two Bovine Mammary Epithelial Cell Lines in 2D and 3D Models. Am J Physiol Cell Physiol.

[32] Zhan K (2017). Three-Dimensional Culture System can Induce Expression of Casein in Immortalized Bovine Mammary Epithelial Cells. Anim Sci J.

[33] Le Jan C (2005). Mammary Transmission of Caprine Arthritis Encephalitis Virus: a 3D Model for in Vitro Study. Reprod Nutr Dev.

[34] Rowson-Hodel AR (2015). Neoplastic Transformation of Porcine Mammary Epithelial Cells in Vitro and Tumor Formation in Vivo. BMC Cancer.

[35] Martignani E (2018). Bovine Mammary Organoids: A Model to Study Epithelial Mammary Cells. Methods Mol Biol.

[36] Zhou Y (2021). Platypus and Echidna Genomes Reveal Mammalian Biology and Evolution. Nature.

[37] Griffiths M, McIntosh D, Coles R (1969). The mammary Gland of the Echidna, Tachyglossus Aculeatus' with Observations on the Incubation of the Egg and on the Newly-Hatched Young. J Zool.

[38] Oftedal OT (2002). The Mammary Gland and Its Origin During Synapsid Evolution. J Mammary Gland Biol Neoplasia.

[39] Hawke T, Bino G, Kingsford RT (2019). A Silent Demise: Historical Insights into Population Changes of the Iconic Platypus (Ornithorhynchus anatinus). Glob Ecol Conserv.

[40] IUCN (2022). Red List of Threatened Species.

[41] Temple-Smith P, Grant T (2001). Uncertain Breeding: a Short History of Reproduction in Monotremes. Reprod Fertil Dev.

[42] Monotreme Resource Center. Available from: https://grutznerlab.weebly.com/monotreme-resource-centre.html.

[43] Neerukonda M (2019). Functional Evaluation of a Monotreme-Specific Antimicrobial Protein, EchAMP, Against Experimentally Induced Mastitis in Transgenic Mice. Transgenic Res.

[44] Kumar A (2019). Structural and Mechanistic Insights into EchAMP: A Antimicrobial Protein from the Echidna Milk. Biochim Biophys Acta Biomembr.

[45] Bisana S (2013). Identification and Functional Characterization of a Novel Monotreme- Specific Antibacterial Protein Expressed During Lactation. PLoS One.

[46] Newman J (2018). Structural Characterization of a Novel Monotreme-Specific Protein with Antimicrobial Activity from the Milk of the Platypus. Acta Crystallogr F Struct Biol Commun.

[47] Enjapoori AK (2014). Monotreme Lactation Protein is Highly Expressed in Monotreme Milk and Provides Antimicrobial Protection. Genome Biol Evol.

[48] Eldridge MDB (2019). An Emerging Consensus in the Evolution, Phylogeny, and Systematics of Marsupials and their Fossil Relatives (Metatheria). J Mammal.

[49] Schep R (2016). Control of Hoxd Gene Transcription in the Mammary Bud by Hijacking a Preexisting Regulatory Landscape. Proc Natl Acad Sci.

[50] Tian R (2022). Molecular Evolution of Vision-Related Genes may Contribute to Marsupial Photic Niche Adaptations. Front Ecol Evol.

[51] Long CA (1969). The origin and Evolution of Mammary Glands. Bioscience.

[52] Oftedal OT (2012). The evolution of Milk Secretion and its Ancient Origins. Animal.

[53] Lincoln DW, Renfree MB (1981). Mammary Gland Growth and Milk Ejection in the Agile Wallaby, Macropus agilis, Displaying Concurrent Asynchronous Lactation. J Reprod Fertil.

[54] Nicholas KR (1988). Asynchronous Dual Lactation in a Marsupial, the Tammar Wallaby (Macropus eugenii). Biochem Biophys Res Commun.

[55] Lemon M, Bailey L (1966). A Specific Protein Difference in the Milk from Two Mammary Glands of a Red Kangaroo. Aust J Exp Biol Med Sci.

[56] Wanyonyi SS (2017). Transcriptome Analysis of Mammary Epithelial Cell Gene Expression Reveals Novel Roles of the Extracellular Matrix. Biochem Biophys Rep.

[57] Wanyonyi SS (2013). The Extracellular Matrix Locally Regulates Asynchronous Concurrent Lactation in Tammar Wallaby (Macropus eugenii). Matrix Biol.

[58] Wanyonyi SS (2013). The Extracellular Matrix Regulates Maeucath1a Gene Expression. Dev Comp Immunol.

[59] Gupta PB (2019). Phenotypic Plasticity: Driver of Cancer Initiation, Progression, and Therapy Resistance. Cell Stem Cell.

[60] Visvader JE (2011). Cells of Origin in Cancer. Nature.

[61] Nolan E, Lindeman GJ, Visvader JE (2023). Deciphering Breast Cancer: from Biology to the Clinic. Cell.

[62] Zhang M, Lee AV, Rosen JM (2017). The Cellular Origin and Evolution of Breast Cancer. Cold Spring Harb Perspect Med.

[63] Luo M, Brooks M, Wicha MS (2015). Epithelial-Mesenchymal Plasticity of Breast Cancer Stem Cells: Implications For Metastasis and Therapeutic Resistance. Curr Pharm Des.

[64] Bouras T (2008). Notch Signaling Regulates Mammary Stem Cell Function and Luminal Cell-Fate Commitment. Cell Stem Cell.

[65] Melchor L (2014). Identification of Cellular and Genetic Drivers of Breast Cancer Heterogeneity in Genetically Engineered Mouse Tumour Models. J Pathol.

[66] Bittner JJ (1936). Some Possible Effects of Nursing on the Mammary Gland Tumor Incidence in Mice. Science.

[67] Ross SR (2010). Mouse Mammary Tumor Virus Molecular Biology and Oncogenesis. Viruses.

[68] Callahan R, Smith GH (2008). The Mouse as a Model for Mammary Tumorigenesis: History and Current Aspects. J Mammary Gland Biol Neoplasia.

[69] Dudley JP, Golovkina TV, Ross SR (2016). Lessons Learned from Mouse Mammary Tumor Virus in Animal Models. ILAR J.

[70] Munson L, Moresco A (2007). Comparative Pathology of Mammary Gland Cancers in Domestic and wild Animals. Breast Dis.

[71] Casey HW, Giles RC, Kwapien RP (1979). Mammary Neoplasia in Animals: Pathologic Aspects and the Effects of Contraceptive steroids. Recent Results Cancer Res.

[72] Rauner G, Ledet MM, Van de Walle GR (2018). Conserved and Variable: Understanding Mammary Stem Cells Across Species. Cytometry A.

[73] Peaker M (2023). Dairy Animals and Breast Cancer: Reflections on a Long-Term Study From the 1970s that was Never Done. J Dairy Res.

[74] Harman RM (2023). miRNA-214-3p Stimulates Carcinogen-Induced Mammary Epithelial Cell Apoptosis in Mammary Cancer-Resistant Species. Commun Biol.

[75] Ledet MM (2018). Differential Signaling Pathway Activation in 7,12-Dimethylbenz[a] Anthracene (DMBA)-Treated Mammary Stem/Progenitor Cells from Species with Varying Mammary Cancer Incidence. Oncotarget.

[76] Harman RM (2021). Beyond Tradition and Convention: Benefits of Non-Traditional Model Organisms in Cancer Research. Cancer Metastasis Rev.

[77] Van Keymeulen A (2011). Distinct Stem Cells Contribute to Mammary Gland Development and Maintenance. Nature.

[78] Davis FM (2016). Single-Cell Lineage Tracing in the Mammary Gland Reveals Stochastic Clonal Dispersion of Stem/Progenitor Cell Progeny. Nat Commun.

[79] Rios AC (2016). The Complexities and Caveats of Lineage Tracing in the Mammary Gland. Breast Cancer Res.

[80] Lloyd-Lewis B (2018). Neutral Lineage Tracing of Proliferative Embryonic and Adult Mammary Stem/Progenitor Cells. Development.

[81] Van Keymeulen A (2017). Lineage-Restricted Mammary Stem Cells Sustain the Development, Homeostasis, and Regeneration of the Estrogen Receptor Positive Lineage. Cell Rep.

[82] Wuidart A (2016). Quantitative Lineage Tracing Strategies to Resolve Multipotency in Tissue-Specific Stem Cells. Genes Dev.

[83] Rios AC (2014). In situ Identification of Bipotent Stem Cells in the Mammary Gland. Nature.

[84] Cereser B (2018). Analysis of Clonal Expansions Through the Normal and Premalignant Human Breast Epithelium Reveals the Presence of Luminal Stem Cells. J Pathol.

[85] Watson C, Kreuzaler P (2011). Remodeling Mechanisms of the Mammary Gland During Involution. Int J Dev Biol.

[86] Chapman RS (1999). Suppression of Epithelial Apoptosis and Delayed Mammary Gland Involution in Mice With a Conditional Knockout Of Stat3. Genes Dev.

[87] Sargeant TJ (2014). Stat3 Controls Cell Death During Mammary Gland Involution by Regulating Uptake of Milk Fat Globules and Lysosomal Membrane Permeabilization. Nat Cell Biol.

[88] Hughes K, Watson CJ (2018). The Multifaceted Role of STAT3 in Mammary Gland Involution and Breast Cancer. Int J Mol Sci.

[89] Li M (1997). Mammary-Derived Signals Activate Programmed Cell Death During the First Stage of Mammary Gland Involution. Proc Natl Acad Sci U S A.

[90] Sharp JA (2006). Fur Seal Adaptations to Lactation: Insights into Mammary Gland Function. Curr Top Dev Biol.

[91] Sharp JA, Lefèvre C, Nicholas KR (2008). Lack of Functional Alpha-Lactalbumin Prevents Involution in Cape Fur Seals and Identifies the Protein As an Apoptotic Milk Factor in Mammary Gland Involution. BMC Biol.

[92] Sharp JA (2017). Dimeric but not Monomeric α-Lactalbumin Potentiates Apoptosis by up Regulation of ATF3 and Reduction of Histone Deacetylase Activity in Primary and Immortalised Cells. Cell Signal.

[93] Durham SD (2023). Creation of a Milk Oligosaccharide Database, MilkOligoDB, Reveals Common Structural Motifs and Extensive Diversity Across Mammals. Sci Rep.

[94] Berger PK (2023). Human Milk Oligosaccharides and Infant Neurodevelopment: A Narrative Review. Nutrients.

[95] Guernsey MW (2017). Molecular conservation of marsupial and eutherian placentation and lactation. ELife.

[96] Stannard HJ, Miller RD, Old JM (2020). Marsupial and Monotreme Milk-a Review of its Nutrient and Immune Properties. PeerJ.

[97] Lefèvre CM, Sharp JA, Nicholas KR (2010). Evolution of Lactation: Ancient Origin and Extreme Adaptations of the Lactation System. Annu Rev Genomics Hum Genet.

[98] Sharp JA (2017). The tammar wallaby: A Marsupial Model to Examine the Timed Delivery and Role of Bioactives in Milk. Gen Comp Endocrinol.

[99] Hester SN (2012). Is the Macronutrient Intake of Formula-Fed Infants Greater Than Breast-Fed Infants in Early Infancy?. J Nutr Metab.

[100] van Leeuwen SS (2019). Challenges and Pitfalls in Human Milk Oligosaccharide Analysis. Nutrients.

[101] Urashima T (2013). Recent Advances in Studies on Milk Oligosaccharides of Cows and Other Domestic Farm Animals. Biosci Biotechnol Biochem.

[102] Asakuma S., et al., Variation of Major Neutral Oligosaccharides Levels in Human Colostrum. Eur J Clin Nutr. 2007. 10.1038/sj.ejcn.1602738 17375110

[103] Kunz C (1999). Lactose-Derived Oligosaccharides in the Milk of Elephants: Comparison With Human Milk. Br J Nutr.

[104] Green B, Merchant J, Newgrain K (1987). Milk Composition in the Eastern Quoll, Dasyurus Viverrinus (Marsupialia:Dasyuridae). Aust J Biol Sci.

[105] Messer M (1987). Changes in Milk Carbohydrates During Lactation in the Eastern Quoll, Dasyurus Viverrinus (Marsupialia). Comp Biochem Physiol B.

[106] Urashima T (2023). Lactose or Milk Oligosaccharide: which is Significant among Mammals?. Anim Front.

[107] German JB (2008). Human Milk Oligosaccharides: Evolution, Structures and Bioselectivity as Substrates for Intestinal Bacteria. Nestle Nutr Workshop Ser Pediatr Program.

[108] Tsugami Y (2020). Establishment of an in Vitro Culture Model to Study Milk Production and the Blood-Milk Barrier with Bovine Mammary Epithelial Cells. Anim Sci J.

[109] Wu RY (2022). Variations in the Composition of Human Milk Oligosaccharides Correlates with Effects on Both the Intestinal Epithelial Barrier and Host Inflammation: A Pilot Study. Nutrients.

[110] Fernández L (2020). The Microbiota of the Human Mammary Ecosystem. Front Cell Infect Microbiol.

